# Strong Bottlenecks Constrain Adaptive Coevolution in a Host–Parasite Metapopulation

**DOI:** 10.1111/mec.70047

**Published:** 2025-07-21

**Authors:** Pascal Angst, Christoph R. Haag, Frida Ben‐Ami, Peter D. Fields, Dieter Ebert

**Affiliations:** ^1^ Department of Environmental Sciences, Zoology University of Basel Basel Switzerland; ^2^ Tvärminne Zoological Station University of Helsinki Hanko Finland; ^3^ CEFE, Université de Montpellier, CNRS, EPHE, IRD Montpellier France; ^4^ School of Zoology George S. Wise Faculty of Life Sciences, Tel Aviv University Tel Aviv Israel

**Keywords:** daphnia, host–parasite interactions, loss of heterozygosity, microsporidia, population structure, runs of homozygosity

## Abstract

Although parasites are well‐known for adaptively evolving in order to exploit their hosts, they may experience strong genetic drift during transmission bottlenecks when infecting a new host. Host population structure and host population bottlenecks can also lead to genetic drift effects in parasite populations, constraining their adaptive evolution further. Here, we investigate the role of host population structure on the evolution of the microsporidian parasite *Hamiltosporidium tvaerminnensis* in a dynamic metapopulation of its microcrustacean host *Daphnia magna* . In following whole‐genome allele frequencies of 59 host and parasite subpopulations for up to 10 years, we found that both antagonists showed patterns of co‐dispersal and isolation‐by‐distance, but allele frequencies were much more dynamic in the host, where we found signatures of recurrent genetic bottlenecks. In the parasite, we observed high levels of shared heterozygosity among subpopulations but also subpopulation‐specific runs of homozygosity (ROHs). We hypothesise that deleterious ROHs, which originate from loss of heterozygosity events during asexual recombination, are fixed in subpopulations following host‐mediated parasite population bottlenecks when host and parasite co‐disperse into a habitat patch where the parasite is not yet present. Thus, host population structure and metapopulation dynamics leave a clearly traceable genomic signature in the coevolving parasite. Contrary to the prevailing assumption that parasites evolve at higher rates than their hosts, our study not only suggests that parasites can evolve more slowly than their host, but also that host dynamics can accelerate drift processes in the parasite.

## 1 Introduction

Parasite evolution is driven by interactions with their hosts and by transmission dynamics between hosts (Buckingham and Ashby 2022). Parasites are known for their high adaptive potential, evolving to exploit host resources, evade host defences and optimise virulence and transmission efficiency through deterministic processes that drive molecular change (Lopez Pascua et al. 2014; Shim and Galvani 2009; Stahl et al. 1999). Stochastic processes may also play a role in parasite evolution, for example when transmission‐related bottlenecks lead to increased genetic drift (Bendall et al. 2023; Bergstrom et al. 1999). In addition, parasite evolution may be influenced by the structure and dynamics of the host population. Host population structure can lead to metapopulation dynamics with frequent extinction and recolonisation in the parasite (Vercken et al. 2010), causing recurrent founder effects and, thus, small effective population sizes and high genetic drift (Polechová and Barton 2015; Whitlock 2004). Thus, host metapopulation dynamics may increase the stochastic processes acting on the host and the parasite population. However, the role of host population dynamics in the evolution of their parasites is not fully understood.

Comparative population genomics provides a way to study population structure and the interplay of deterministic and stochastic evolutionary processes among populations of different species (Edwards et al. 2022). Although originally designed for phylogenetically related species, this comparative approach allows us to study the evolution of phylogenetically unrelated but sympatric species simultaneously by unravelling differences in their respective evolutionary trajectories. The causes of these differences can then be attributed to specific aspects of the species' biology—their ecology, their life history, their mode of reproduction (Delmotte et al. 1999; Edwards et al. 2022). Comparative population genomics is most promising for species in long‐term intimate associations, as in symbionts who rely on a single host species for survival and reproduction (Moran and Plague 2004). By comparing a host and its parasite, this approach can assess the effect of a given feature in one species, for example, their particular ecology, on a population genomic estimate of both species, for example, population structure. For example, McCoy et al. (2005) found that the parasitic tick *Ixodes uriae* is more strongly structured than its seabird host, *Rissa tridactyla* , which they attributed to the host's within‐breeding season movement. van Schaik et al. (2014) studied two parasitic mite species, each infecting a different bat species and found different levels of population structure, genetic drift and gene flow in the parasites, attributing this difference to variation in host social systems. Although these studies demonstrate the potential of genetics to compare multiple species, their conclusions have been mostly limited to population structure. Here we take the next step, using high‐throughput whole‐genome sequencing to investigate the interplay of microevolutionary processes in shaping the evolution of a host and its parasite across time and space.

The sympatric occurrence of the microcrustacean *Daphnia magna* and its microsporidian parasite *Hamiltosporidium tvaerminnensis* in well‐studied metapopulations offers the opportunity to examine the impact and interaction of these species' ecologies, life histories and demographies on the (co)evolutionary process. The pond‐dwelling *D. magna* occurs in distinct water bodies, forming distinguishable subpopulations suitable for population genomics (Ebert 2022). High environmental variation among its habitat patches enables us to discern potential ecological impacts on evolutionary dynamics even at small spatial scales. Moreover, despite sharing the same environment, host and parasite have different modes of reproduction, cyclical parthenogenetic and asexual respectively, with potentially different gene flow rates that can influence their evolutionary dynamics differentially. A long‐term study of a *D. magna* metapopulation across the Baltic Skerry islands in southwestern Finland, where more than 50% of all subpopulations are infected with *H. tvaerminnensis*, suggested high turnover dynamics with severe genomic consequences (Angst et al. 2024; Dubart et al. 2020; Ebert et al. 2001, 2013; Pajunen 1986; Pajunen and Pajunen 2003). Frequent extinction and (re)colonisation by small numbers of founders led to low effective population sizes, strong genetic drift, weak purifying selection and low rates of adaptive evolution in *D. magna* (Angst, Ameline, et al. 2022; Haag et al. 2005, 2006). Consistently, the evolution of resistance to *H. tvaerminnensis* is largely impeded in this *D. magna* metapopulation (Cabalzar et al. 2019). However, compared to uninfected subpopulations, infected host subpopulations were found to have weak selection signatures at several genetic regions involved in resistance and tolerance for this parasite (Halter et al. 2022; Krebs et al. 2017) and a slightly reduced genetic load (Cabalzar et al. 2019). On the Skerry islands of the Tvaerminne archipelago, Southwestern Finland, the fungi‐related intracellular parasite *H. tvaerminnensis* exclusively infects *D. magna* via mixed‐mode transmission—both horizontally (from decaying host cadavers) and vertically (through (a)sexual host eggs) (Haag et al. 2011, 2013). A molecular study of 16 *H. tvaerminnensis* genes found an excess of (fixed) heterozygote polymorphisms, a lack of recombination and longer than expected coalescence times, indicative of asexual reproduction (Haag et al. 2013).

In this population genomic study, we aim to understand how *D. magna* metapopulation dynamics affect its parasite, *H. tvaerminnensis*. Specifically, we ask whether the population genetic structure of *H. tvaerminnensis* matches that of the host metapopulation, which would suggest co‐dispersal (Mazé‐Guilmo et al. 2016). We further ask whether and how the *D. magna* population structure and evolutionary dynamics affect the evolution of *H. tvaerminnensis*, especially genetic drift, which is strong in the small subpopulations of its host and has led to the accumulation of deleterious mutations (Angst, Ameline, et al. 2022). To address these questions, we apply pooled co‐sequencing to a metapopulation with over 550 habitat patches to characterise the spatial genomic variation of host and parasite. Repeatedly sampling the whole‐genome allele frequencies of its subpopulations over a 10‐year period allows us to investigate population genomic patterns and potential changes in these patterns. We might further be able to quantify deterministic and stochastic evolutionary processes and compare the impact and interaction of these processes on shaping host and parasite evolution.

## 2 Methods

### 2.1 Study System and Samples

**FIGURE 1:**
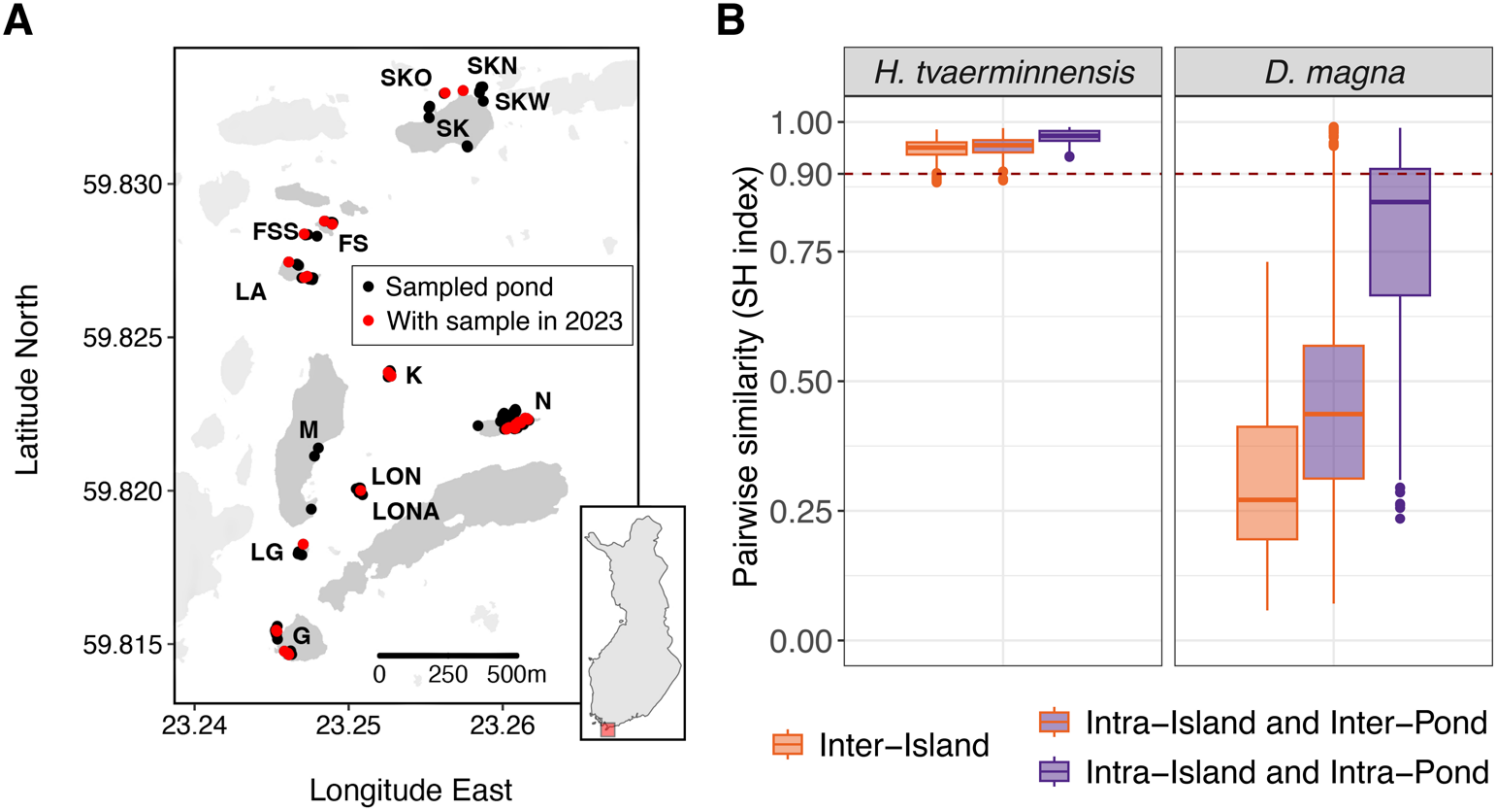
Geographic locations of sampled ponds and estimated pairwise genetic similarity of the inhabiting subpopulations. (A) The map shows the study islands with dots indicating the geographic locations of sampled ponds and red dots representing ponds sampled in 2023. The study area contains about five times more habitat patches (ponds), but *D. magna* did not occur there during the study period. Island names and abbreviations were adopted from the long‐term study (see e.g., Angst, Ameline, et al. 2022). The inset map shows Finland with the study area marked with a red square. Islands shown in light grey are not part of the biannual census and were, therefore, not sampled. (B) Pairwise genetic similarity, SH index, is much higher between parasite samples (left) than between host samples (right) and mostly above 0.9. Within each species, SH is highest for temporal subpopulation samples from the same pond (right‐most box in each graph) and slightly higher for spatially and/or temporally separated samples from the same island (middle box) than for those from different islands (left box). The boxplots show the median, 25th and 75th percentiles, whiskers extending to the minimum and maximum values within 1.5 times the 25th and 75th percentiles, respectively and data points outside this interval as dots.

This study uses natural population samples of the planktonic freshwater crustacean *Daphnia magna* and its microsporidian parasite *Hamiltosporidium tvaerminnensis*. We collected the samples on the Tvaerminne archipelago in southwestern Finland (59°50′ N, 23°15′ E; Figure 1A), where *D. magna* populations inhabit shallow rock pools. More than half of these populations are naturally infected with *H. tvaerminnensis* (Ebert et al. 2001). Subpopulations are connected by gene flow, forming a metapopulation with extinction–(re)colonisation dynamics. In both host and parasite, gene flow and colonisation occur mostly via the passive dispersal of *D. magna* resting stages by wind, water and birds. Created by sexual reproduction, these resting stages usually hatch early in the season or after rain refills dehydrated rock pools; they allow the cyclical parthenogen to survive inhospitable conditions (Ebert 2022). The parasite *H. tvaerminnensis* is transmitted horizontally and vertically from one host to the next, surviving as a resting spore inside or outside of the host's resting stage. *Hamiltosporidium tvaerminnensis* reproduces asexually, and hosts in which *Hamiltosporidium* spp. have been suggested to reproduce sexually are absent in this habitat (Haag et al. 2013; Vizoso et al. 2005). More than 550 rock pools on the Tvaerminne archipelago have been surveyed biannually for *D. magna* since 1982, and their infection with *H. tvaerminnensis* and other, less common parasites has been checked since 2007 (Dubart et al. 2020; Ebert et al. 2013; Pajunen 1986; Pajunen and Pajunen 2003). Between 2014 and 2018, whole‐genomic sequencing of pooled individuals (pool‐seq) was performed for each occupied rock pool with Illumina paired‐end reads using HiSeq 2500 and NovaSeq 6000 sequencers as part of a spatiotemporal study (Angst et al. 2024). Here, rock pools are referred to as ponds so as not to confuse the term with genomic pool‐seq samples.

For this study, we reused the genomic paired‐end pool‐seq samples of the *D. magna* metapopulation mentioned above (NCBI database; Bioproject ID: PRJNA862292; Angst et al. (2024)). Pool‐seq provides estimates of genome‐wide allele frequencies of populations that are largely comparable to individual‐based sequencing, at lower cost and in less time (Gautier et al. 2013; Schlötterer et al. 2014). However, genetic inferences are limited to the population level when pool‐seq is used because it is unclear from which individual each DNA or sequencing read originates. Samples were taken in late May/early June (= spring samples) and late July/August (= summer samples) from 2014 to 2018 and consisted of approximately 50 *D. magna* pooled per subpopulation and time point before DNA extraction. In 2023, additional spring samples were collected from 29 subpopulations continuously occupied by *D. magna* . These samples were processed and sequenced in the same manner (Figure 1A, Table S1). We determined the presence of *H. tvaerminnensis* in the samples based on field records and the whole‐genome read depth estimates obtained from mapping the sequencing reads to the *H. tvaerminnensis* reference genome.

### 2.2 Variant Calling Pipeline

To obtain variant calls, we first trimmed the pool‐seq reads to remove low‐quality sequences and adapter contamination using Trimmomatic v.0.39 (Bolger et al. 2014) and interleaved trimmed reads using seqtk v.1.2 mergepe (https://github.com/lh3/seqtk). All bioinformatical software was set at default settings unless stated otherwise. The snakemake (Mölder et al. 2021) workflow described in Angst, Ameline, et al. (2022) (https://github.com/pascalangst/Angst_etal_2022_MBE) was used to map interleaved reads separately to a host and a parasite reference genome and to produce a VCF file for each organism. For the host, this was the XINB3 individual genome (10 chromosomes in 603 contigs; genome size: 141.4 Mb; NCBI database; Assembly name: ASM4014379v1; GenBank assembly accession: GCA_040143795.1; BioProject ID: PRJNA624896; Cornetti et al. (2024)) and for the parasite, this was the FI‐OER‐3‐3 isolate near‐chromosomal genome (11 chromosomes in 17 scaffolds; genome size: 21.6 Mb; NCBI database; Assembly name: FIOER33 v3; GenBank assembly accession: GCA_022605425.2, BioProject ID: PRJNA778105; Angst et al. (2023)). Both reference genomes stem from the Tvaerminne archipelago and are therefore closely related to the study samples. All calculations and average read depth estimations for both reference genomes using SAMtools function depth were performed at sciCORE (http://scicore.unibas.ch/) scientific computing center at the University of Basel. In the VCF files, we used VCFtools v.0.1.16 (Danecek et al. 2011) and BCFtools v.1.9 (Danecek et al. 2021) to mask entries with depth of coverage (DP) less than ten and larger than twice the mode and VcfFilterJdk v.1f97a34 (Lindenbaum and Redon 2018) to mask entries with allele depth (AD) of the minor allele equal to one. For that, we calculated DP mode separately for each sample based on the sum of the ADs. As co‐sequencing of host and parasite resulted in a reduced number of sequencing reads per organism, we excluded samples with sequence data for less than 95% of all variable sites in the VCF files. Finally, we retained only biallelic high quality SNP variants (QUAL > 30, MQ > 40, QD > 2.0, MAC > 1, FS < 60) present in at least 90% of all samples for analysis. Sample filtering was done using the R v.4.0.3 (R Core Team 2020) package VCFR v.1.12.0 (Knaus and Grünwald 2017), while variant filtering used vcffilter from the C++ library vcflib v.1.0.0_rc2 (Garrison 2019), VCFtools and VcfFilterJdk.

### 2.3 Population Genetic Analyses

To check for asexual parasite lineages in the metapopulation, we estimated genomic similarity by calculating the shared heterozygosity (SH) index (Yu et al. 2023) for each sample pair using BCFtools. To compare, we calculated SH indices across host samples. The SH index is calculated from non‐missing genotype calls by dividing the number of shared heterozygote positions by the number of heterozygote positions of the sample with the highest number of heterozygote positions. After observing SH outliers in comparisons including *H. tvaerminnensis* samples with many non‐reference homozygous positions and few heterozygous positions, we identified runs of homozygosity (ROH) in *H. tvaerminnensis* samples, excluding repetitive regions of the genome, sites with missing genotypes and sites with uncertain genotype calls (GQ < 99) using plink v.1.90b6.18 (Chang et al. 2015) with the parameters ‐‐homozyg‐snp 10 and ‐‐homozyg‐kb 10, following guidelines for robust ROH analysis (Meyermans et al. 2020). We then applied all population genetic analyses to the full genome of host and parasite and separately to chromosomal scaffolds without ROHs of the parasite.

To estimate spatial population structure and test for isolation‐by‐distance (IBD), we calculated pairwise *F*_ST_ of samples from summer 2015 (supplemented by summer 2014 samples for subpopulations without available summer 2015 sample) using poolfstat v.2.2.0 (Gautier et al. 2022). These cross‐sections had the most *H. tvaerminnensis* genomic samples because the parasite is most prevalent in summer and thus better represented in the sequencing reads, and because the number of ponds occupied by host subpopulations decreased across the sampled years. Testing for IBD included decomposing the pairwise *F*_ST_ matrix into principal components using the R base stats function prcomp(), calculating geographic distances between sampling locations using the geodist v.0.0.8 (Padgham and Sumner 2019) R package, transforming geographic distances to a distance‐based Moran's eigenvector map (dbMEM) using the adespatial v.0.3–23 (Dray et al. 2021) R package, and redundancy analysis (RDA) to explain variation in genomic differentiation with the dbMEM as implemented in the vegan v.2.6–4 (Oksanen et al. 2020) R package with significance assessed by 1000 permutations. Similarly, we assessed the relationship between pairwise genomic differentiation of host and parasite using samples with data available for both at this timepoint.

To visually represent population structure, we estimated effective migration surfaces (EEMSs) as implemented in runeems_snps (Petkova et al. 2016) using standardised Euclidean distances between samples calculated from their genome‐wide allele frequencies. EEMSs highlight potential regions of higher‐than‐average and lower‐than‐average historic gene flow between habitat patches. Because of the non‐continuous distribution of our species, we generated a custom population grid with each habitat patch connected to its five closest neighbours to represent mostly local dispersal and connected to one other random patch to account for uncommon long‐distance dispersal. Both dispersal types have been suggested for this metapopulation (Angst et al. 2024; Dubart et al. 2020; Pajunen 1986; Pajunen and Pajunen 2003). We used the spatial analysis samples for PCA and all samples for t‐SNE (t‐distributed stochastic neighbour embedding) clustering based on whole‐genome allele frequencies as implemented in the pcadapt v.4.4.0 (Privé et al. 2020) and Rtsne v.0.17 (Krijthe 2015) R packages with 5D retained from the initial PCA step, perplexity 16 and 5000 iterations. We further used the pairwise *F*_ST_ matrix of the spatial analysis samples for generating neighbour‐nets with SplitsTree v.6.4.11 (Huson and Bryant 2024) and a tanglegram of neighbour‐joining trees with the ape v.5.7–1 (Paradis and Schliep 2019) R package. Finally, to plot temporal dynamics, we transformed pairwise *F*_ST_ between all samples of the same pond using ln((*F*_ST_ + 0.001)/(1−(*F*_ST_ + 0.001))) and fitted a nonlinear model described by *F*_ST_ = *ab* ^1/∂t^, where ∂t is the time between samples that was previously used for temporal description of pairwise *F*_ST_ (Angst et al. 2024; Bergland et al. 2014). For visual representation, we used R packages of tidyverse (Wickham et al. 2019), Dendroscope v.3.8.10 (Huson and Scornavacca 2012), reemsplots2 v.0.1.0 (Petkova et al. 2016) and a shapefile from Ranta10, Finish Environment Institute (2021).

## 3 Results

### 3.1 Samples and (Un)Shared Heterozygosity

Using a previously compiled spatiotemporal genomic dataset that collected and co‐sequenced 418 samples of *D. magna* and non‐bacterial symbionts from 118 ponds between 2014 and 2018 (Angst et al. 2024, See Methods) and an additional 29 genomic samples from 29 ponds collected in 2023 (total samples = 447), we found greater than 1× coverage of the *H. tvaerminnensis* genome in about two‐thirds (*n* = 308) of the samples from about three‐quarters of all ponds (*n* = 88). The relative number of infected subpopulations was stable over the study period. One hundred sixty parasite samples (36%) from 59 ponds (50%) remained after filtering the genomic data, each with greater than 20× average coverage for the *H. tvaerminnensis* genome. From these samples, we obtained 170,710 biallelic, high‐quality SNPs. For the host genome assembly, which is about seven times larger, 849,472 biallelic sites passed the same filters. We used these filtered datasets for subsequent genomic analyses.

To determine genetic similarity within each species, we calculated the SH index (Yu et al. 2023) separately between all sample pairs of the likely asexual *H. tvaerminnensis* and between all sample pairs of the cyclic parthenogenetic *D. magna* . We found that most heterozygote sites were shared across parasite samples (Figure 1B), with most similarity estimates being above the proposed threshold of 0.9 for detecting intraclone samples (Yu et al. 2023). This suggests fixed heterozygosity across parasite individuals likely due to asexual reproduction in *H. tvaerminnensis* and hence low genetic variation among subpopulations. Fixed heterozygosity is further supported by allele frequencies of around 50% in all parasite subpopulation samples at these heterozygous sites. Of the observed similarity values lower than 0.9 in the parasite, at least one sample came from island LA (Sample IDs: LA‐16_smr2015 and LA‐20_smr2015), which had the most non‐reference homozygous positions but the fewest heterozygous positions of all the samples. As expected for a cyclic parthenogenetic organism, *D. magna* 's shared heterozygosity was much lower (Figure 1B). Only a few host subpopulation samples showed high genetic similarity, especially those from the same or geographically very close subpopulations and the same year. This might be because *D. magna* experiences strong founder bottlenecks, subsequent inbreeding, and asexual reproduction between samples from the same year (Angst, Ameline, et al. 2022).

**FIGURE 2:**
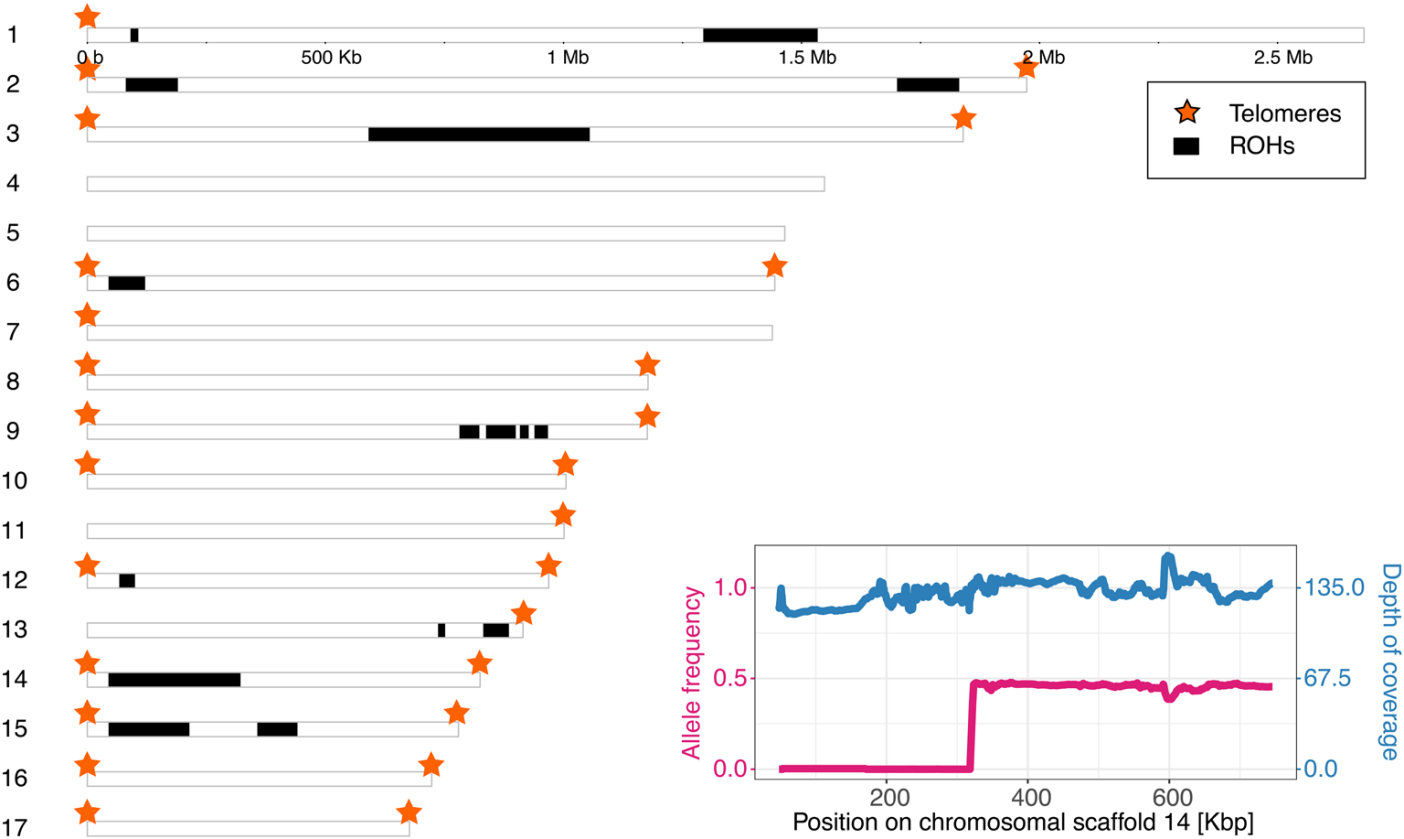
Parasite ROHs are found in a subset of subpopulations but they are positioned across the genome. The karyoplot of *H. tvaerminnensis* shows all 17 scaffolds, of which 11 represent complete chromosomes with two telomeres (Angst et al. 2023). The inset shows an example of an ROH from the LA‐16 subpopulation on scaffold 14. Alternative alleles at polymorphic sites have a frequency of about 0.5 across the genome, except in some regions of some subpopulations where they have a frequency of 0, that is, in ROH regions. In these regions, the depth of sequencing coverage is about the genome‐wide average. Lines represent rolling means of 100 positions.

Homozygous sites in parasite samples from LA island, which were heterozygous in samples from other islands, had sequencing read coverages similar to the genome‐wide average of these samples. Still, only one allele was represented in the reads. These sites, clustered in the *H. tvaerminnensis* genome, indicate a switch from heterozygosity to homozygosity in larger genomic regions of the LA samples. Given previous study of Scandinavian *H. tvaerminnensis* suggests that our parasite metapopulation originated from the spread of a single asexual lineage (Angst, Ebert, and Fields 2022), these homozygous regions represent runs of homozygosity (ROHs) originating from loss of heterozygosity (LOH) events. Among the 160 samples from 59 ponds, we identified ROHs in a total of 23 samples (15%) from 11 ponds (20%). 19 ROHs were greater than 10 kilobase pairs (Kbp), including 9 ROHs greater than 100 Kbp (Figure 2, Table S2). Most ROHs were unique to individual parasite subpopulations but were shared among temporal samples of the same subpopulation, except one size‐polymorphic ROH from pond SK‐58, which was, however, identical in the first and the last sample of this pond. In pond N‐71, where we observed local extinction and subsequent recolonisation of *D. magna* and the parasite, the newly founded parasite subpopulation did not have the ROHs observed in the previously extinct subpopulation. The SH index of the two parasite samples from island LA were among the lowest (0.887) because they had the most and longest ROHs (LA‐20_smr2015: 5 ROHs and LA‐16_smr2015: 1 ROH of 465 Kbp). Excluding scaffolds with ROHs in at least one of all samples from the calculation of the SH index, which was about two‐thirds of all scaffolds, yielded a similar value for the LA samples (0.974) as for samples from the same pond.

### 3.2 Population Structure

**FIGURE 3:**
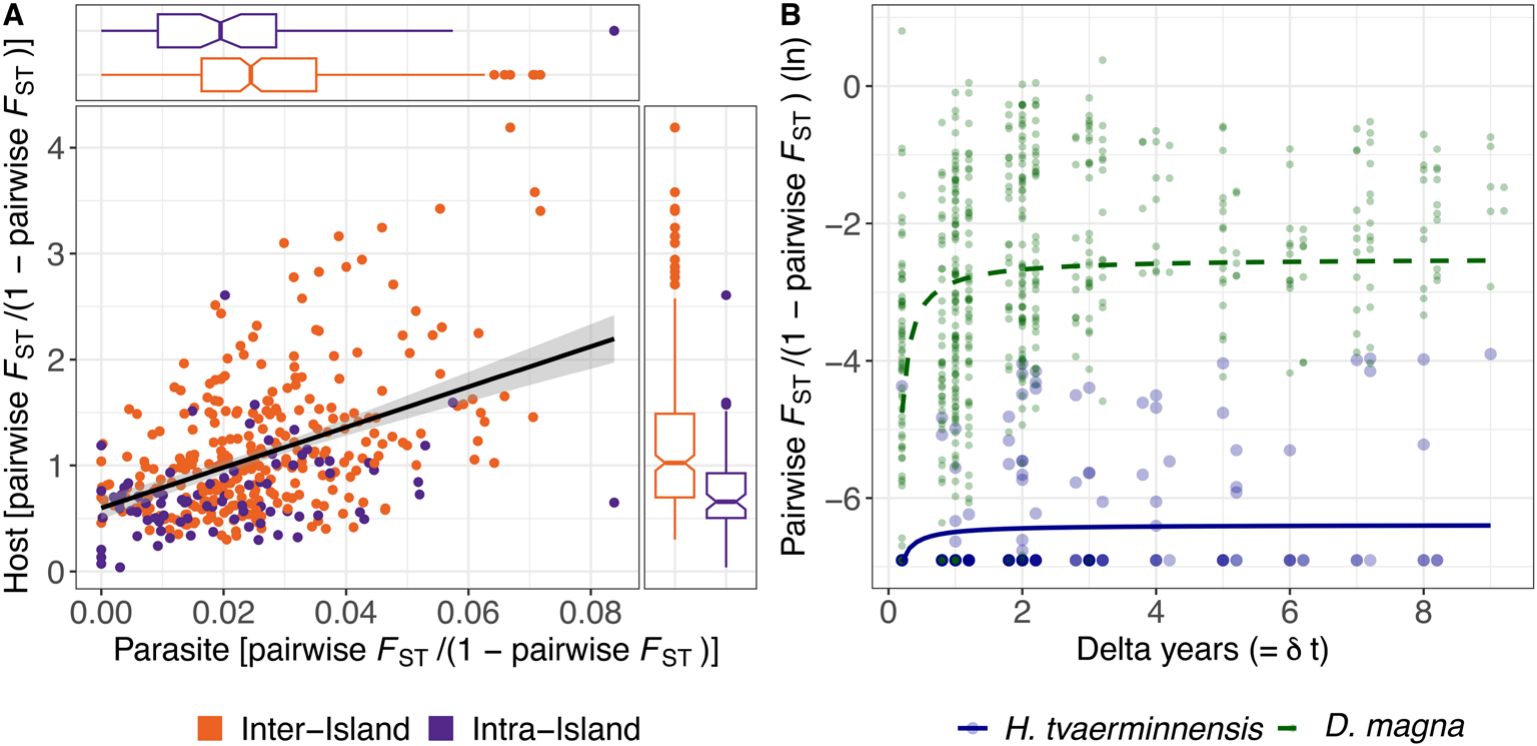
Pairwise estimates of allele frequency variation between host and parasite subpopulations. (A) Host and parasite pairwise *F*_ST_ are positively correlated and pairwise *F*_ST_ is higher between subpopulations from different islands (inter‐island) than between subpopulations from the same island (intra‐island). The black regression line with the grey 95% confidence interval is from a linear model to illustrate the positive correlation between host and parasite spatial variation in allele frequencies. The boxplots show the median, 25th and 75th percentiles, whiskers extending to 1.5 times the 25th and 75th percentiles, respectively and data points outside this interval as dots. Notches indicate the 95% confidence interval of the median. (B) Genome‐wide variation in allele frequencies among temporally separated samples from the same pond measured as pairwise *F*_ST_ is generally very low in the parasite (large dots, bold line) and high in the host (small dots, dashed line). Lines represent predicted *F*_ST_ values based on nonlinear regression (*F*_ST_ = ab^1/∂t^) as used by Bergland et al. (2014). The minimum ∂t value corresponds to the time between our spring and summer samples.

To investigate the parasite's spatial population structure, we reduced temporal effects by including only one host–parasite sample per subpopulation from summer 2015 (*n* = 27) or, when no sample from summer 2015 was available, from the preceding summer (*n* = 9). The combined samples from these two time points covered the full geographic range where the parasite occurred. Using pairwise *F*_ST_, we found a positive correlation between pairwise *F*_ST_ and geographic distance for the parasite (dbMEM analysis by RDA: *R* ^2^ = 0.28, *p* = 0.006, *n* = 36), which has previously been reported for the *D. magna* host using the same approach (Angst, Ameline, et al. (2022)). This indicates IBD in both host and parasite. Considering only parasite samples from the same island, we found, in contrast to the host (Angst, Ameline, et al. (2022)), no statistical correlation between pairwise *F*_ST_ and geographic distance (e.g., island N: *R* ^2^ = 0.16, *p* = 0.575, *n* = 17) unless we excluded scaffolds with ROHs (*R* ^2^ = 0.40, *p* = 0.032, *n* = 17). Variation in host allele frequencies between subpopulations from different islands was higher than between subpopulations from the same island (Wilcoxon: *W* = 6643, *p* = 5.761 × 10^−11^; Figure 3A). The parasite showed the same pattern but at a smaller scale and with a smaller difference (*W* = 9631.5, *p* = 0.001), indicating less pronounced spatial population structure. Host pairwise *F*_ST_ was positively correlated with parasite pairwise *F*_ST_ (*R* ^2^ = 0.30, *p* = 0.019; *n* = 28; Figure 3A), but it was substantially lower in the parasite (mean pairwise *F*_ST_ = 0.024) than in the host (0.482; Figure 3A). Using the full spatiotemporal dataset, we further found low temporal variation in allele frequencies in *H. tvaerminnensis*, again in contrast to the host (Figure 3B; Angst et al. (2024)). In the host, high genome‐wide spatiotemporal variation in subpopulation allele frequencies has been shown to be driven by metapopulation dynamics and bottlenecks, causing strong genetic drift (Angst et al. 2024). In contrast, low genome‐wide spatiotemporal variation in subpopulation allele frequencies in the parasite likely reflects a past bottleneck, followed by exclusive asexual reproduction. In both cases, however, natural selection is strongly impeded.

**FIGURE 4:**
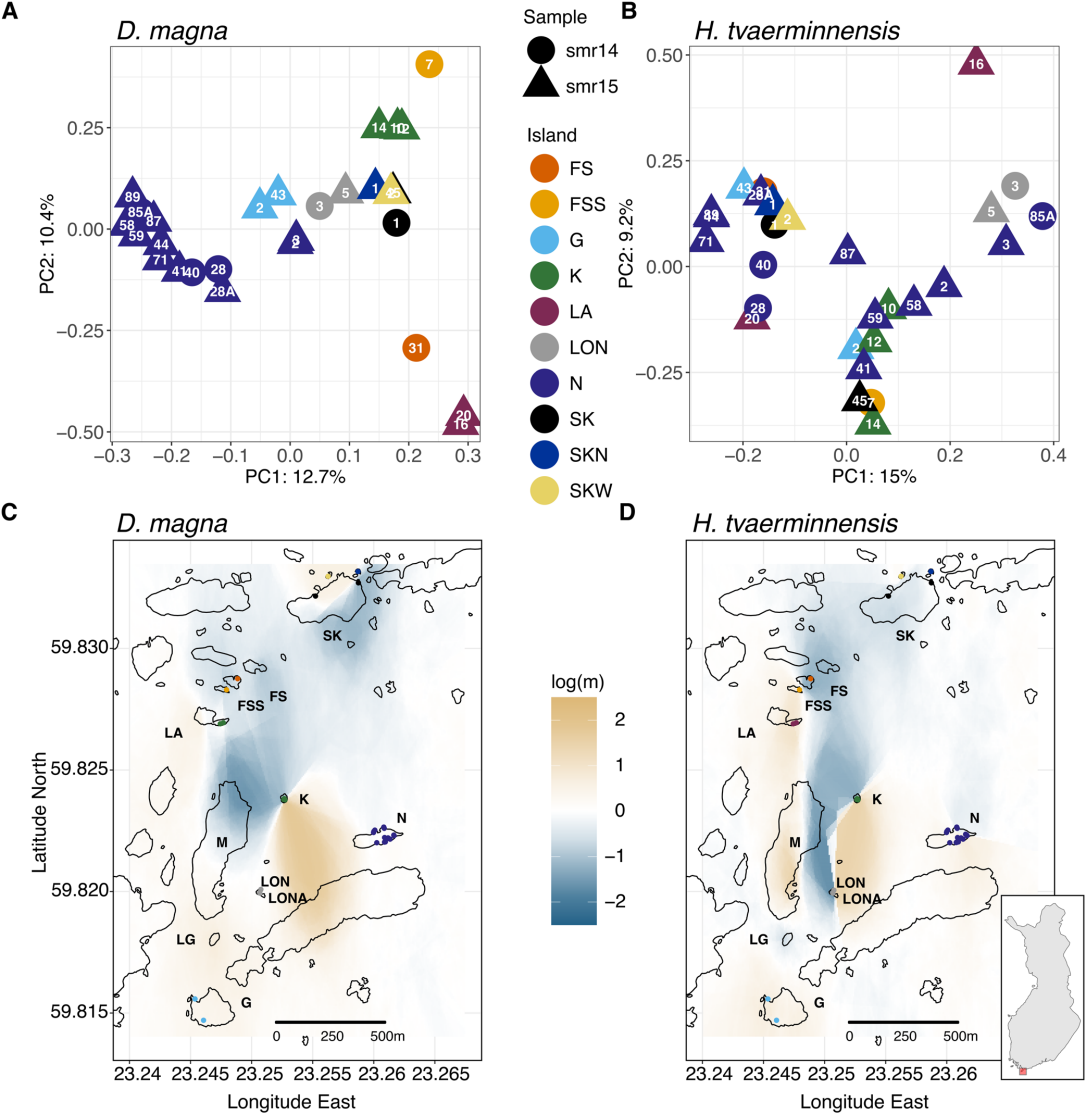
Population structure estimated using PCA and visualised as estimated effective migration surfaces (EEMSs). Dimensionality‐reduction of host (A) and parasite (B) genetic samples using PCA, with coloured symbols representing the island of origin, sample time and pond ID number. Percentages indicate the variance explained by PC1 and PC2. The heatmap reflects estimated higher‐than‐average (positive log (m)) and lower‐than‐average (negative log (m)) historical gene flow in the host (C) and the parasite (D). The map shows the study islands with dots indicating the geographic locations of samples used in the analysis. The inset map shows Finland with the study area indicated with a red square.

We estimated principal components (PCs) and effective migration surfaces to visually depict population structure. The former clusters subpopulations by genetic distance while the latter estimates potential geographic regions of higher‐than‐average and lower‐than‐average historical gene flow from the genetic distances and geographic locations of subpopulations. PCA showed that samples from the same island tended to cluster in the host (Figure 4A) and in the parasite (Figure 4B), consistent with our finding of IBD in the parasite and previous findings of IBD and clustering by island for the host metapopulation (Angst et al. 2024). Samples did not cluster by time of sample collection. Host and parasite EEMSs showed a similar geographic pattern, but estimated gene flow was overall more evenly distributed in the parasite than the host (Figure S1), reflecting the parasite's lower overall spatial variation in allele frequencies between subpopulations (Figure 3A). As this approach uses genetic distances averaged across the entire genome, parasite ROHs contribute disproportionally to its outcome because SNPs and ROHs most likely originate from single mutation events but alter allele frequencies differently (a ROH mutation affects many sites). To investigate historical gene flow based only on SNP mutations, independent of the ROHs and thereby avoiding this bias, we generated an EEMS using scaffolds of the parasite without ROHs (Figure 4C,D). As expected, based on the correlation of their genetic population structure (Figure 3A), this parasite EEMS showed the same regions of lower‐ and higher‐than‐average historical gene flow as the host EEMS. Although the dispersal dynamics in this system are not fully understood, previous studies have shown that both species are passively dispersed by wind, water and birds (Altermatt and Ebert 2010; Zumbrunn 2011), the activities of which may explain the observed historical gene flow patterns.

Taken together, our population structure findings for the two species are consistent with co‐dispersal of host and parasite. Our field observations are also consistent with co‐dispersal: during the first 5 years of dense sampling, 19 (43%) of the 44 observed, newly established host subpopulations were infected. Furthermore, of the four parasite immigrations that we observed into parasite‐free subpopulations, one coincided with gene flow into the host subpopulation (Pond ID: G‐2; Angst et al. (2024)). In the other three cases, no host gene flow was detected during the study time, either because the likely co‐dispersed host immigrants may have not (yet) become sufficiently frequent due to priority effects, or because the parasite was dispersed separately. Co‐dispersal is not surprising given that, in addition to transmitting horizontally, the parasite also transmits vertically through the host's sexual eggs, which serve as host resting and dispersal stages.

### 3.3 Level of Co‐Differentiation

**FIGURE 5:**
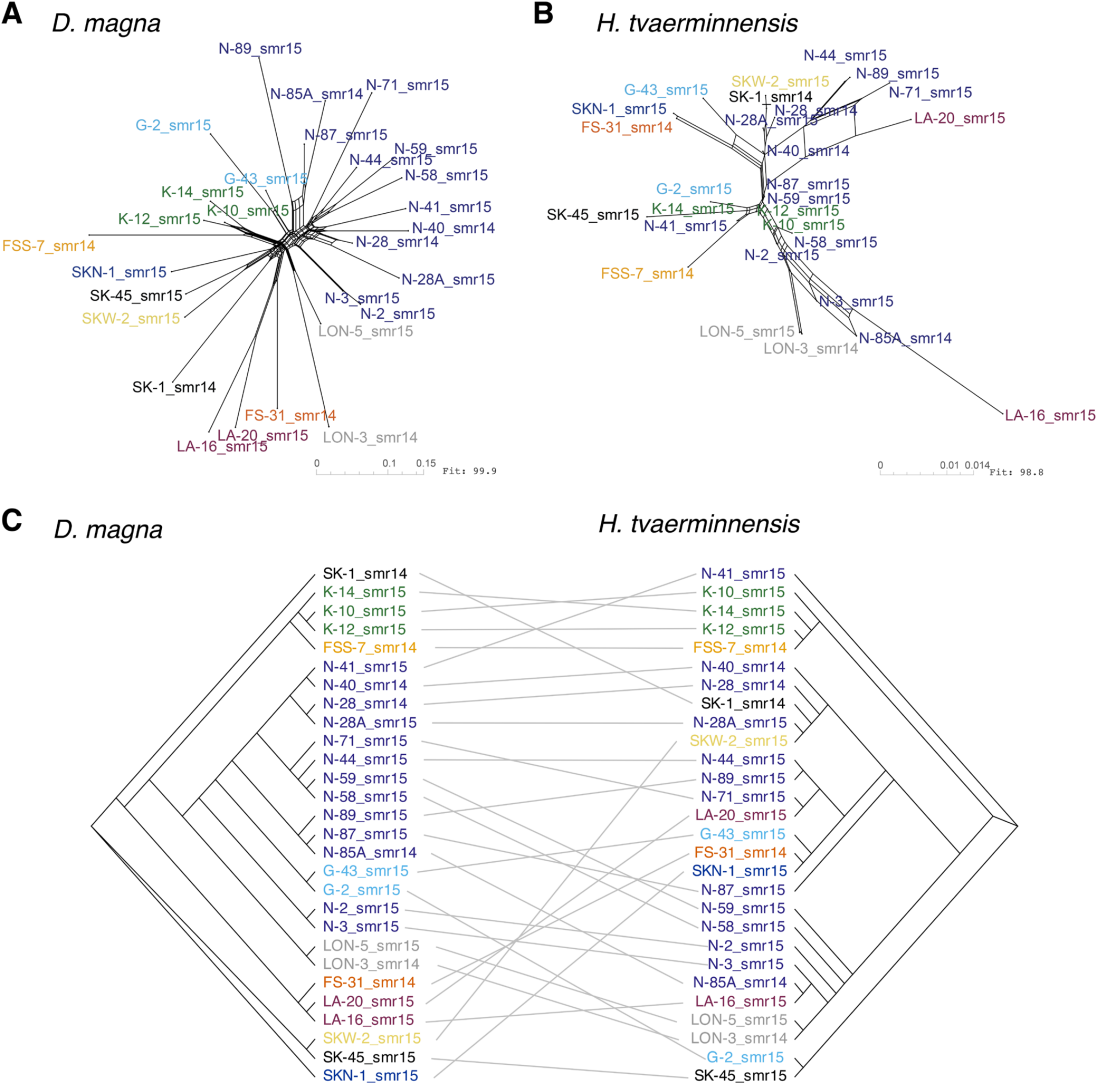
Genetic relationships of host and parasite subpopulations displayed as neighbour‐nets and a tanglegram. Split networks show genetic relationships separately between host (A) and parasite (B) subpopulations. (C) The tanglegram opposes host (left) and parasite (right) neighbour‐joining trees, which show simplified but similar genetic relationships as the neighbour‐nets. The neighbour‐nets and trees are based on pairwise *F*_ST_ calculated from whole‐genome allele frequencies. Subclades roughly encompass samples from the same ponds and islands. Colours are reused from Figure 4.

Since the shared host–parasite population structure is likely a consequence of co‐dispersal, we expected a pattern of co‐differentiation, with exceptions when one antagonist fails to establish. We investigated this by applying clustering based on whole‐genome allele frequencies and neighbour‐joining tree estimation. Dimensionality reduction to two dimensions using t‐SNE clustering of all parasite subpopulation samples (including temporal samples) showed that samples from the same pond tended to cluster (Figure S2A), which is consistent with our finding of low temporal variation in allele frequencies of individual parasite subpopulations (Figure 3B). An exception was pond N‐71, where we observed local extinction and subsequent recolonisation of *D. magna* . Also, samples from the same island generally, but not always, clustered together. For example, samples from islands LA and SK did not cluster, unless scaffolds with ROHs were excluded (Figure S2B). Genetic clustering by pond and island has previously been reported for this host metapopulation (Angst et al. 2024), with known exceptions including samples from island FSS, which we also identified here as an exception in the parasite. The clustering of FSS‐7 host and parasite subpopulation samples with samples from island K suggests a long‐distance co‐dispersal event. Generally, although our observation of similar clustering in host and parasite further supports co‐dispersal, neighbour‐joining clustering based on the pairwise *F*_ST_ matrix revealed no strict co‐differentiation of host and parasite (Figure 5). This might partly be because the high similarity of parasite samples complicates inferences about their relationships and clear identification of gene flow and its source. For example, the many similar samples from island N were spread across the parasite clusters. A partial co‐differentiation may be caused by the dispersal dynamics of *D. magna* and *H. tvaerminnensis*, which involves simultaneous (co‐dispersal) and successive dispersal (parasite dispersal after the host). Importantly, while our allele frequency data allow us to cluster similar subpopulations and infer approximate relationships between them, the resulting neighbour‐net and tree‐based visualisations are simplifications of the actual demographic history.

## 4 Discussion

By co‐sequencing hosts and parasites from a natural *D. magna* –*H. tvaerminnensis* metapopulation, we sought to understand the population structure of the two species and the eco‐evolutionary processes that shaped their genetic diversity. Despite their different modes of reproduction, the cyclic parthenogenic host and the asexual parasite both show a population genomic pattern of IBD, which is consistent with co‐dispersal of the parasite with the host. Recurrent colonisation‐related host population bottlenecks affect genetic variation in both species. In the parasite, genetic bottlenecks are reflected in the frequent occurrence of subpopulation‐specific runs of homozygosity (ROH) with only a few subpopulations sharing the same ROH. Parasite variation in genetic diversity was otherwise low across time and space, which is in line with asexual reproduction. Our study demonstrates the intricate way in which host and parasite population structure and dynamics are interwoven, and provides a microevolutionary picture of the evolution in a dynamic host–parasite metapopulation.

### 4.1 Host and Parasite Population Structure, Co‐Dispersal and Coevolution

In our focal *D. magna* metapopulation, colonisation follows the propagule model based mainly on the passive dispersal of resting stages (Slatkin 1977; Zumbrunn 2011). Spatial distribution of *H. tvaerminnensis* suggests its propagules are either dispersed together with the resting stages of infected hosts or separately in the form of waterborne transmission stages. The latter is likely to occur over very short distances, as water exchange between ponds is limited, and could rarely occur over longer distances when birds ingest freshwater containing spores and then egest them in another pond. The dispersal of *D. magna* and *H. tvaerminnensis* can therefore be simultaneous (by co‐dispersal) or successive, when the parasite arrives in a habitat patch already colonised by the host. This dispersal dynamic and its effects on infection patterns and population genetic structure have been shown in the specific human pathogen *Helicobacter pylori* (Moodley et al. 2009): While the host can colonise all available habitat patches, the obligate parasite is constrained to either disperse with the host or to colonise habitat patches already occupied by the host. Because the host might spread more rapidly, newly founded *D. magna* subpopulations are often parasite‐free (Ebert et al. 2001), representing a form of parasite escape (Pajunen and Pajunen 2007). Unless these hosts are resistant, these new subpopulations might subsequently become infected and remain so (Lass and Ebert 2005). An experimental study of field‐collected individuals demonstrated that genetically diverse host populations evolve in response to parasite treatment (Zbinden et al. 2008); however, the evolution of resistance seems to be strongly impeded by colonisation‐related population bottlenecks, strong genetic drift and low efficiency of natural selection in this *D. magna* metapopulation (Angst, Ameline, et al. 2022; Cabalzar et al. 2019; Halter et al. 2022).

Consistent with co‐dispersal, the observed host and parasite population genetic structures follow a pattern of IBD. The detected partial co‐differentiation suggests additional parasite dispersal after the host, as has been observed here and during previous longitudinal screening of the metapopulation (Ebert et al. 2001). In this successive host–parasite dispersal scenario, infected host immigrants may introduce parasites into an existing subpopulation but may remain rare themselves, explaining the dissociation between host and parasite genotypes. The pattern of IBD extends up to a continental scale in both host and parasite (Angst, Ebert, and Fields 2022; Fields et al. 2018). Such large‐scale co‐structure has been observed in other host–parasite systems, for example, in *Silene latifolia* and its pathogen *Microbotryum lychnidis‐dioicae* (Feurtey et al. 2016). Existing knowledge about *H. tvaerminnensis*' natural history and the dispersal dynamics of its host therefore helped us better interpret the parasite's population structure, colonisation history and gene flow, but also determine models that fit its observed population genomic patterns. Our findings for the parasite's population structure, for example, that geographically proximate samples are less differentiated than more distant samples, are consistent with the propagule pool model of colonisation but not with the migrant pool model (Slatkin 1977). These findings are in line with those of a previous study on the host metapopulation (Angst et al. 2024). In the propagule pool model, new subpopulations are derived from founders from single subpopulations, whereas in the migrant pool model they are formed by founders from a random sample of all subpopulations.

The similarities between host and parasite population structure indicate a form of coevolution that does not require reciprocal adaptation but is driven by stochastic processes arising from the overlapping ecology of host and parasite. In particular, due to their co‐dispersal, host metapopulation dynamics and strong colonisation bottlenecks affect both antagonists, leading to an entirely neutral coevolutionary process. Similarly, a shared host–parasite evolutionary history associated with co‐dispersal has been recovered from the nematode parasite *Wuchereria bancrofti* and its human host (Small et al. 2019). Human parasites also share the out‐of‐Africa bottleneck and other demographic signatures as a result of co‐dispersal with the host (Doyle et al. 2022; Thorpe et al. 2022). Despite the similar patterns of host and parasite population structure here, the parasite had lower pairwise *F*_ST_ estimates and a smaller difference in inter‐ versus intra‐island pairwise *F*_ST_ than the host (Figure 2).

A limiting factor in our system is the low level of nucleotide diversity in the parasite, which reduces our ability to infer its population structure and demography. Also, because the detected ROHs, unlike SNPs, affect many sites, care needs to be taken in the interpretation of the results. By excluding scaffolds with ROHs from the analysis, we were able to buttress our inferences, but this reduced the overall amount of genetic variation even further. ROHs represent notable differences between the otherwise highly similar parasite samples and can therefore be informative of the evolutionary process. However, the evolutionary significance of the previously unrecognised ROHs in *H. tvaerminnensis* remains to be determined.

### 4.2 Runs of Homozygosity

Most parasite subpopulations showed fixed heterozygosity signatures across the entire genome. However, about 20% showed–mostly subpopulation‐specific–regions of homozygosity. Assuming that shared heterozygosity occurs because all parasite samples originated from a single parasite lineage, these runs of homozygosity likely resulted from independent loss of heterozygosity events, especially as ROHs of different subpopulations were mostly non‐overlapping and on different scaffolds. With the available pool‐seq data, we can only detect ROHs if they are fixed or nearly fixed at the subpopulation level; we cannot detect them in a single individual or at low frequencies. Our sampling, thus, finds a new ROH only after it replaces its heterozygous ancestor or after it occurs in the founder of a new parasite subpopulation. ROHs might disappear from a subpopulation when a parasite genotype without the given ROHs enters the subpopulation and outcompetes the resident parasite, or–in the context of the here employed methods–reaches detection levels. In our temporal sampling, subpopulation‐level ROHs neither appeared nor disappeared in any of the monitored subpopulations, indicating that ROH turnover is a slow process.

The fixation of ROHs can be driven by selective and stochastic processes. Although the functional implications of the ROH are unclear here, the former seems unlikely given our data, as fixation by selection would indicate that ROHs are beneficial and would therefore accumulate and be shared by many subpopulations. That the ROHs did not accumulate suggests instead that they are deleterious, unmasking deleterious mutations from their recessive state, a process akin to inbreeding. It has been postulated that ROHs may lead to a cost that outweighs the two‐fold cost of sexual reproduction, depending on how frequently loss of heterozygosity happens and on how many recessive deleterious mutations there are (Archetti 2010, 2023). If ROHs are deleterious, parasites with ROH do not increase within subpopulations through selective processes in competition with ROH‐free parasites. Instead, we suggest that the ROHs observed here were fixed in association with population bottlenecks during the founding of a new subpopulation, that is, when ROHs are present in the founder propagule of the parasite or when the parasite undergoes loss of heterozygosity during its phase in the host sexual resting stage. After that, these locally established parasites with ROH may have a low chance of colonising other subpopulations because most subpopulations are already infected (about 75% in this study), because they remain infected (Lass and Ebert 2005), and because ROH‐free resident parasites may not allow immigrants with ROH to establish. Since the average age of a subpopulation in this metapopulation is low (about 7 years; Ebert et al. (2001)) and host populations experience parasite immigration only about every 5 years (Ebert et al. 2001), opportunities for a parasite with ROH to spread, except for the occasional establishment in an uninfected host population, seem very low.

Ignoring the apparently rare events where a non‐ROH parasite replaces an ROH parasite (which we never observed), the observation that 20% of all infected subpopulations have ROHs that mostly arose independently suggests that the *de novo* rate of heterozygosity loss is rather high and may even be underestimated because ROHs are removed by selection from subpopulations with ROH‐free parasites (Boyer et al. 2021). Furthermore, if ROHs are mostly fixed by genetic bottlenecks, genetic bottlenecks must be frequent in the parasites of this metapopulation. This aligns with the evidence for recurrent bottlenecks in the host. *D. magna* experiences bottlenecks during the (re)colonisation of empty ponds, often with a bottleneck size of 1 (Haag et al. 2002). Subsequent selfing leads to severe inbreeding depression, with 50% of heterozygote sites becoming homozygote (Cornetti et al. 2021). Host and parasite bottlenecks are likely wedded by their co‐dispersal, which coincides with our field observations, the biology of the system and the correlated host and parasite population genetic features, that is, a coevolutionary process. Taken together, ROHs seem to be produced recurrently in the parasite and are locally fixed by population bottlenecks but can only persist for limited time periods. These bottlenecks likely co‐occur with host bottlenecks, stressing how host structure and demography influence parasite evolution.

### 4.3 Potential Mechanisms of the Evolution of ROHs in Microsporidia

Loss of heterozygosity can be caused by recombination associated with sexual or asexual reproduction. Sexual reproduction (with complete meiosis) leads to frequent (dis)appearance of ROHs across the genome and a quick erosion of fixed heterozygosity unless sex is very rare (Hartfield 2021; Ho et al. 2019). In asexual organisms, mechanisms to erode (and maintain) heterozygosity like non‐meiotic recombination or incomplete meiosis are crucial for long‐term existence (Blanc et al. 2023; Dukić et al. 2019; Laine et al. 2022). Examples of such recombination include gene conversion, double‐strand breaks with subsequent mismatch repair leading to the replacement of a chromosomal segment by its homologue, and abortive meiosis, where, for example, in yeast fungi, mitotic division is initiated after crossover, or, in the crustacean *Artemia*, one meiotic division is suppressed (Bast et al. 2018; Boyer et al. 2021; Mozzachiodi et al. 2021; Smukowski Heil 2023). The shorter ROHs we observed might originate from gene conversion, while larger ROHs close to the chromosomal scaffold ends might originate from mismatch repair or abortive meiosis (Table S2). Although the latter ROHs are expected to extend to the chromosome tips, which our observed ROHs did not, some sequencing reads may have been mis‐mapped in these difficult‐to‐align genetic regions that had above‐average coverage, introducing artificial heterozygosity to the ROH regions. Mis‐mapping could also explain the variation in the SK‐58 subpopulation ROH length, as this ROH overlapped with a repeat‐rich genetic region. Recombination during asexual reproduction, which is common in (partially) asexual species including fungal species like yeasts (James et al. 2019), ascomycetes (Schoustra et al. 2007) and oomycetes (Dale et al. 2019), has been suggested but never shown for the microsporidia (Corradi 2015). Altogether, our findings of almost genome‐wide shared heterozygosity among the parasite samples, ROHs in some subpopulations' chromosomal scaffolds, and the absence of a second host on which *Hamiltosporidium* spp. could rely upon for sexual reproduction (Mangin et al. 1995) are most parsimoniously explained by asexual reproduction with non‐meiotic recombination or abortive meiotic recombination.

## 5 Conclusion

Our results, along with previous findings (Angst, Ameline, et al. 2022; Angst, Ebert, et al. 2022; Haag et al. 2020; Halter et al. 2022), suggest that the adaptive evolution of host resistance and parasite virulence in this natural *D. magna* –*H. tvaerminnensis* metapopulation system is strongly constrained and that their coevolution is better explained by a shared ecology leading to correlated stochastic changes in genetic diversity. In the host, extinction–(re)colonisation dynamics lead to recurrent founder effects and, thus, high genetic drift with strong and stochastic shifts in genome‐wide allele frequencies and, concomitantly, inefficient natural selection. In the parasite, genome‐wide heterozygosity seems to be fixed, or at least little changed since a historical switch from sexual to asexual reproduction during the geographical expansion to the Northern Eurasian range. Locally restricted loss of heterozygosity events indicate strong bottlenecks—likely through founder events, a characteristic of the host's metapopulation dynamics—that amplify the effects of the low effective population sizes in this parasite, leading to even more reduced selection efficacy than has been observed across its continental distribution. Although likely to be mostly deleterious, LOH is a form of large‐scale genetic mutation that may facilitate the adaptation and evolution of virulence in asexually reproducing microsporidia, like shown in pathogenic *Candida* and other yeast fungi (Mba et al. 2022; Smukowski Heil 2023). Despite providing insights into loss of heterozygosity events in microsporidia, understanding their cause and role in microsporidia evolution requires additional research.

Our study showcases a scenario in which host and parasite coevolution is dominated by stochastic effects. Nevertheless, adaptive processes are visible, for example, selection against ROHs in the parasite, as they are mostly not shared among subpopulations, and heterozygote advantage in the host (Angst et al. 2024). The cyclic parthenogenetic host appears to evolve faster than the parasite, while at the same time accelerating stochastic evolution in the parasite through the mediated population bottlenecks, countering conventional wisdom that parasites have faster rates of adaptative evolution than their host. Parasite population bottlenecks associated with host metapopulation dynamics have also been shown in parasitic smut fungi of the herb *Silene* (Delmotte et al. 1999; Vercken et al. 2010). Further studies are needed to determine if the frequency and strength of parasite bottlenecks are lower in host populations without metapopulation dynamics, where stochastic effects in the host are weaker (Fields et al. 2018). Our study demonstrates the importance of knowing the life history and demography of an organism to correctly infer the processes shaping its evolution. This knowledge combined with progress in high‐throughput sequencing can be applied to understand evolutionary interactions between species and, thus, to gain insight into the rate and mode of (co)evolution in sympatric species.

## Author Contributions

P.A., P.D.F. and D.E. designed the study. P.A. analysed the data. P.A., C.R.H., F.B.‐A., P.D.F. and D.E. conducted the field work. F.B.‐A. and D.E. identified infections in the long‐term metapopulation survey. D.E. supervised the long‐term metapopulation data collection and curated the data. P.A., P.D.F., D.E. and F.B.‐A. collected and P.A. and P.D.F. prepared the metapopulation samples. P.A. wrote the manuscript with input from all authors. All authors reviewed and approved the final manuscript.

## Conflicts of Interest

The authors declare no conflicts of interest.

## Supporting information


Data S1.

## Data Availability

Analysis scripts are available at https://github.com/pascalangst/Angst_etal_2025_MolEcol, raw data are deposited at the NCBI SRA database (BioProject IDs PRJNA862292 and PRJNA1134700).
