# Supplementary material for: Strong Bottlenecks Constrain Adaptive Coevolution in a Host–Parasite Metapopulation
Source: Mol Ecol. 2025 Jul 21;34(17):e70047. doi: 10.1111/mec.70047 (PMC12376958; doi:10.1111/mec.70047)

**Supplemental Information for:**

**Strong bottlenecks constrain adaptive coevolution in a host–parasite metapopulation**

Pascal Angst^1,2,*^, Christoph R. Haag^2,3^, Frida Ben-Ami^2,4^, Peter D. Fields^1,2,†^,
and Dieter Ebert^1,2,†^

^1^ Department of Environmental Sciences, Zoology, University of Basel,
Basel 4051, Switzerland

^2^ Tvärminne Zoological Station, University of Helsinki, Hanko 10900, Finland

^3^ CEFE, Université de Montpellier, CNRS, EPHE, IRD, Montpellier 34293, France

^4^ School of Zoology, George S. Wise Faculty of Life Sciences, Tel Aviv University,
Tel Aviv 6997801, Israel

^*^ Corresponding author: [pascal.angst@unibas.ch](mailto:pascal.angst@unibas.ch)

^†^ Shared last author

**Table S1: 2023 sample information and mapping statistics.** Sample names (IDs) are formed by the pond name and the sampling date. They are accompanied by the number of pooled animals prior to DNA extraction (#animals), the percentage of sequencing reads that mapped to the *D. magna* reference genome (mapping_percent), the average whole-genome coverage (average_coverage), and the *H. tvaerminnensis* infection status (infection_status). Details of samples from 2014 to 2018 are available from Angst et al. (2024).

| ID | #animals | mapping_percent | average_coverage | infection_status |
| --- | --- | --- | --- | --- |
| FS-27_spr2023 | 48 | 0.9874 | 115.022 | 0 |
| FS-3_spr2023 | 50 | 0.9903 | 167.901 | 0 |
| FSS-18_spr2023 | 37 | 0.9869 | 320.338 | 0 |
| G-10_spr2023 | 47 | 0.9918 | 332.234 | 0 |
| G-42_spr2023 | 50 | 0.8918 | 337.08 | 1 |
| G-43_spr2023 | 50 | 0.8402 | 251.24 | 1 |
| G-45_spr2023 | 50 | 0.9896 | 282.992 | 0 |
| G-5_spr2023 | 50 | 0.9816 | 294.068 | 1 |
| K-10_spr2023 | 50 | 0.9861 | 102.72 | 1 |
| K-14_spr2023 | 47 | 0.9824 | 331.748 | 1 |
| K-16_spr2023 | 50 | 0.9883 | 301.6 | 1 |
| K-8_spr2023 | 50 | 0.9664 | 83.2921 | 1 |
| LA-10_spr2023 | 50 | 0.9092 | 284.105 | 1 |
| LA-29_spr2023 | 50 | 0.9809 | 335.26 | 0 |
| LA-9_spr2023 | 50 | 0.9925 | 207.16 | 0 |
| LG-1_spr2023 | 50 | 0.8006 | 235.11 | 1 |
| LON-1_spr2023 | 39 | 0.9481 | 61.2951 | 1 |
| N-26_spr2023 | 50 | 0.9868 | 234.408 | 1 |
| N-27_spr2023 | 50 | 0.9882 | 193.028 | 1 |
| N-28_spr2023 | 46 | 0.9831 | 284.146 | 1 |
| N-41_spr2023 | 32 | 0.9691 | 205.105 | 0 |
| N-42_spr2023 | 50 | 0.9914 | 142.412 | 0 |
| N-46_spr2023 | 50 | 0.82 | 203.72 | 1 |
| N-50_spr2023 | 35 | 0.983 | 59.8916 | 1 |
| N-61_spr2023 | 43 | 0.9674 | 85.1658 | 1 |
| N-68_spr2023 | 50 | 0.7426 | 375.612 | 1 |
| N-71_spr2023 | 37 | 0.6876 | 173.315 | 1 |
| SK-58_spr2023 | 38 | 0.8999 | 430.099 | 1 |
| SKW-2_spr2023 | 40 | 0.6728 | 294.073 | 1 |

**Table S2: ROHs greater than 10 Kbp and their location in the genome.** Pond ID is a combination of the island and a consecutive number. Sampling timepoint is a combination of the season and year of sampling. Sample and locations details can be found in Angst et al. (2024). Scaffolds are near-chromosomal level.

| **Pond ID** | **Timepoint** | **Scaffold** | **Scaffold length (bp)** | **Start (bp)** | **End (bp)** | **Length (Kbp)** |
| --- | --- | --- | --- | --- | --- | --- |
| FS-31 | smr2014 | scaffold_1 | 2,681,470 | 1,293,872 | 1,533,679 | 239.81 |
| FS-31 | smr2017 | scaffold_1 | 2,681,470 | 1,293,872 | 1,533,679 | 239.81 |
| FSS-7 | smr2014 | scaffold_15 | 779,687 | 44,516 | 214,064 | 169.55 |
| FSS-7 | spr2014 | scaffold_15 | 779,687 | 44,516 | 214,064 | 169.55 |
| G-2 | smr2015 | scaffold_12 | 969,012 | 66,938 | 100,289 | 33.35 |
| G-2 | spr2015 | scaffold_12 | 969,012 | 66,938 | 100,289 | 33.35 |
| LA-16 | smr2015 | scaffold_3 | 1,839,609 | 590,484 | 1,055,301 | 464.82 |
| LA-16 | smr2015 | scaffold_13 | 915,172 | 736,337 | 751,250 | 14.91 |
| LA-16 | smr2015 | scaffold_14 | 824,917 | 44,281 | 321,594 | 277.31 |
| LA-20 | smr2015 | scaffold_9 | 1,176,229 | 781,628 | 823,362 | 41.74 |
| LA-20 | smr2015 | scaffold_9 | 1,176,229 | 837,282 | 899,954 | 62.67 |
| LA-20 | smr2015 | scaffold_9 | 1,176,229 | 908,633 | 927,062 | 18.43 |
| LA-20 | smr2015 | scaffold_9 | 1,176,229 | 939,113 | 967,424 | 28.31 |
| LA-20 | smr2015 | scaffold_15 | 779,687 | 357,270 | 441,231 | 83.96 |
| N-19 | spr2018 | scaffold_1 | 2,681,470 | 90,858 | 107,280 | 16.42 |
| N-19 | spr2018 | scaffold_6 | 1,444,059 | 44,396 | 121,484 | 77.09 |
| N-19 | spr2018 | scaffold_13 | 915,172 | 831,480 | 885,268 | 53.79 |
| N-71 | smr2014 | scaffold_2 | 1,973,229 | 80,509 | 189,972 | 109.46 |
| N-71 | smr2014 | scaffold_15 | 779,687 | 357,270 | 441,231 | 83.96 |
| N-71 | smr2015 | scaffold_2 | 1,973,229 | 80,509 | 189,972 | 109.46 |
| N-71 | smr2015 | scaffold_15 | 779,687 | 357,270 | 441,231 | 83.96 |
| N-71 | spr2016 | scaffold_2 | 1,973,229 | 80,509 | 189,972 | 109.46 |
| N-71 | spr2016 | scaffold_15 | 779,687 | 357,270 | 441,231 | 83.96 |
| N-85A | smr2014 | scaffold_3 | 1,839,609 | 590,484 | 892,093 | 301.61 |
| SK-45 | smr2014 | scaffold_2 | 1,973,229 | 1,700,800 | 1,831,404 | 130.61 |
| SK-45 | smr2015 | scaffold_2 | 1,973,229 | 1,700,800 | 1,831,404 | 130.61 |
| SK-45 | smr2016 | scaffold_2 | 1,973,229 | 1,700,800 | 1,831,404 | 130.61 |
| SK-58 | smr2016 | scaffold_3 | 1,839,609 | 590,484 | 772,726 | 182.24 |
| SK-58 | smr2017 | scaffold_3 | 1,839,609 | 590,484 | 745,353 | 154.87 |
| SK-58 | smr2018 | scaffold_3 | 1,839,609 | 642,382 | 772,726 | 130.35 |
| SK-58 | spr2015 | scaffold_3 | 1,839,609 | 590,484 | 772,726 | 182.24 |
| SK-58 | spr2016 | scaffold_3 | 1,839,609 | 604,143 | 772,726 | 168.58 |
| SK-58 | spr2023 | scaffold_3 | 1,839,609 | 590,484 | 772,726 | 182.24 |
| SKN-1 | smr2015 | scaffold_1 | 2,681,470 | 1,293,872 | 1,533,679 | 239.81 |

**Figure S1: EEMSs based on the full genomic dataset.** A) shows the same as Figure 4C and B) shows the same as Figure 4D but uses genome-wide summarization for estimation.


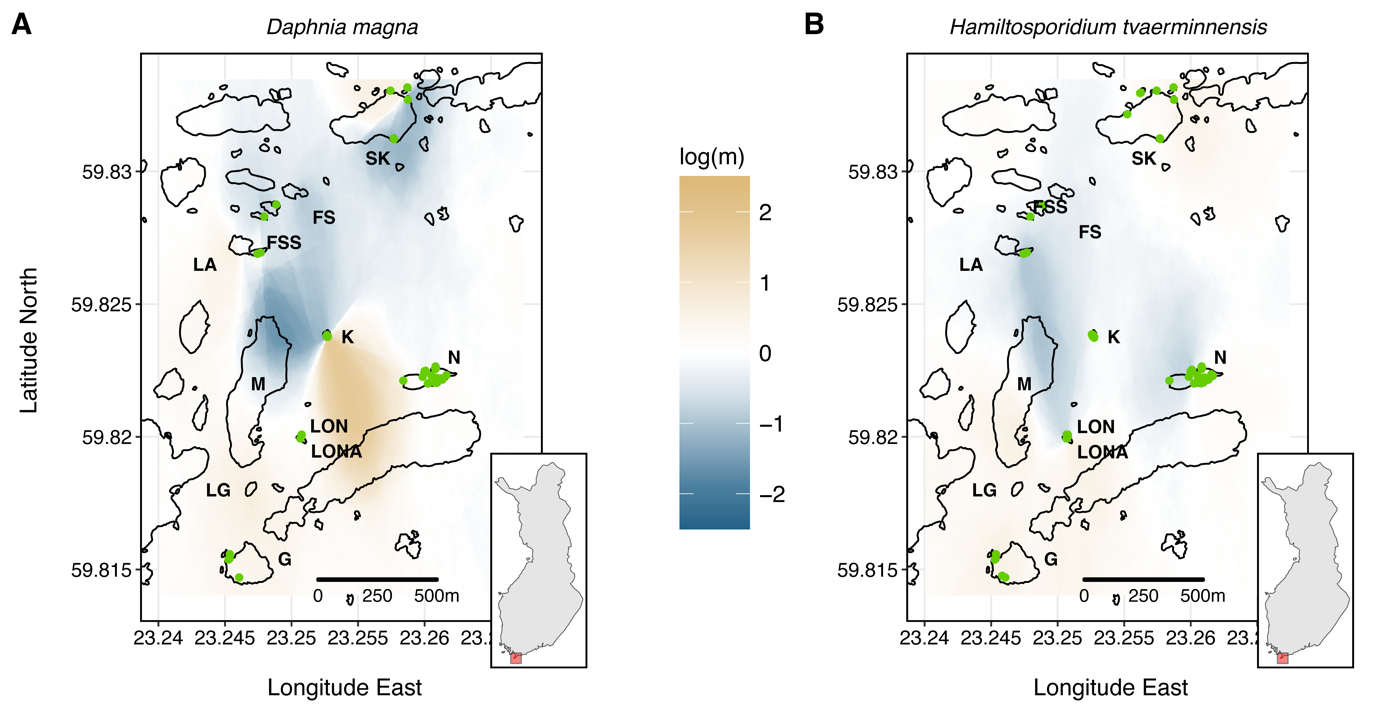


**Figure S2: Dimensionality-reduction of parasite whole-genome allele frequencies (A) and allele frequencies of scaffolds without ROHs (B) using t-SNE.** Clustering reflects the spatial population structure of the parasite metapopulation, which is similar to the host metapopulation structure (Angst et al., 2024) but less clear. For example, samples from island N are broadly distributed, which could be related to low parasite genetic diversity. Additionally, mutations of large effect like the loss of heterozygosity in a larger genetic region could introduce stochasticity, as evidenced by samples with long or many ROHs that do not cluster as expected based on their geographic origin in A), but cluster in B) (e.g., island LA and island SK samples). Shape and colour of symbols indicate the island of origin (see legend).


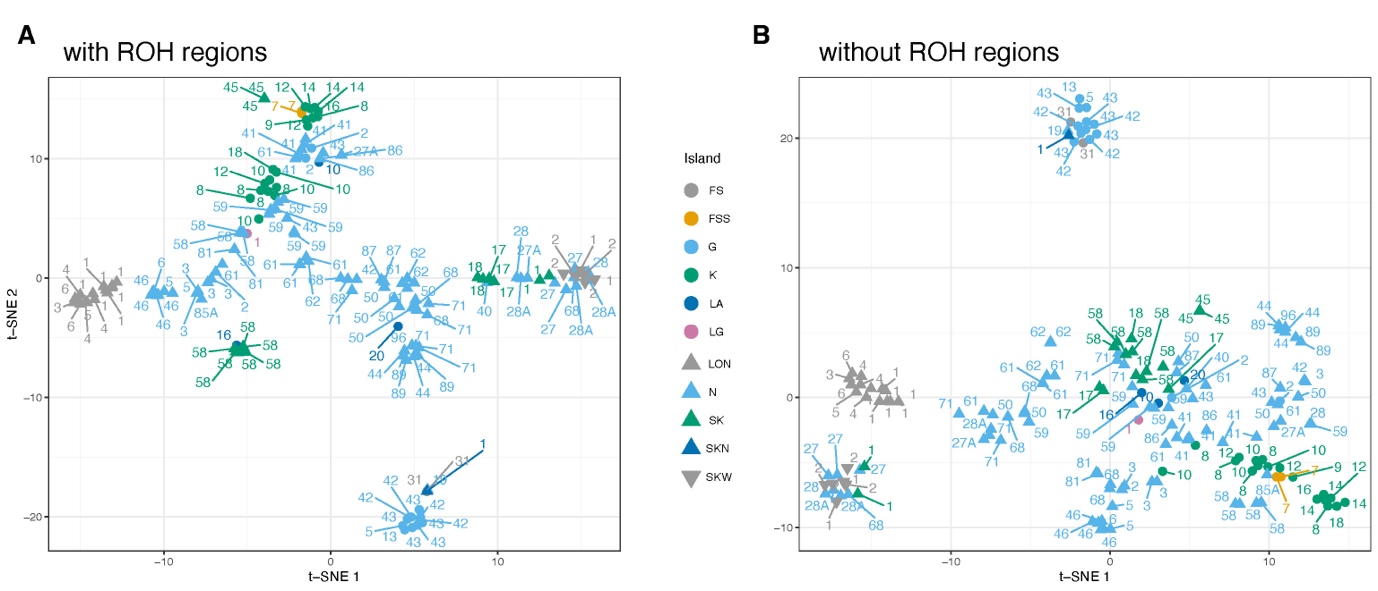

Supplement: Supplementary file 1 — Data S1. [file MEC-34-e70047-s001.docx]
